# Distribution of functional groups in periodic mesoporous organosilica materials studied by small-angle neutron scattering with in situ adsorption of nitrogen

**DOI:** 10.3762/bjnano.3.49

**Published:** 2012-05-30

**Authors:** Monir Sharifi, Dirk Wallacher, Michael Wark

**Affiliations:** 1 Institute of Physical Chemistry and Electrochemistry, Leibniz University Hannover, Callinstr. 3A, D-30167 Hannover, Germany; 2 Laboratory of Industrial Chemistry, Ruhr-University Bochum, Universitaetsstr. 150, D-44801 Bochum, Germany; 3 Berlin Neutron Scattering Center (BENSC), Helmholtz-Zentrum Berlin für Materialien und Energie GmbH, Hahn-Meitner-Platz 1, D-14109 Berlin, Germany

**Keywords:** contrast matching, crystal-like periodicity, distribution of functional groups, PMO, SANS, surface functionalization

## Abstract

Periodic mesoporous materials of the type (R′O)_3_Si-R-Si(OR′)_3_ with benzene as an organic bridge and a crystal-like periodicity within the pore walls were functionalized with SO_3_H or SO_3_^−^ groups and investigated by small-angle neutron scattering (SANS) with in situ nitrogen adsorption at 77 K. If N_2_ is adsorbed in the pores the SANS measurements show a complete matching of all of the diffraction signals that are caused by the long-range ordering of the mesopores in the benzene-PMO, due to the fact that the benzene-PMO walls possess a neutron scattering length density (SLD) similar to that of nitrogen in the condensed state. However, signals at higher *q*-values (>1 1/Å) are not affected with respect to their SANS intensity, even after complete pore filling, confirming the assumption of a crystal-like periodicity within the PMO material walls due to π–π interactions between the organic bridges. The SLD of pristine benzene-PMO was altered by functionalizing the surface with different amounts of SO_3_H-groups, using the grafting method. For a low degree of functionalization (0.81 mmol SO_3_H·g^−1^) and/or an inhomogeneous distribution of the SO_3_H-groups, the SLD changes only negligibly, and thus, complete contrast matching is still found. However, for higher amounts of SO_3_H-groups (1.65 mmol SO_3_H·g^−1^) being present in the mesopores, complete matching of the neutron diffraction signals is no longer observed proving that homogeneously distributed SO_3_H-groups on the inner pore walls of the benzene-PMO alter the SLD in a way that it no longer fits to the SLD of the condensed N_2_.

## Introduction

Regarding functionalization with organic groups, Si-MCM-41 often suffers from pore blocking at the pore mouths and inhomogeneous distribution of the functional groups in the case of post-synthetic grafting [1–3] or, if the co-condensation route is employed, from the loss of the mesoporous structure due to a lack of optimum micelle formation with the increase in the organic/functional loadings [4]. In addition the wide application of grafting for the modification of Si-MCM-41 materials is hindered due to the lack of reactive centers apart from the OH-groups (silanol groups) present on the inner surface [3]. Therefore periodic mesoporous organosilicas (PMOs) have attracted much attention in scientific and technological research since their discovery in 1999 by three independent groups [5–7]. PMO materials directly form hybrid organic–inorganic matrices, as they are built from precursors of the type (R′O)_3_Si-R-Si(OR′)_3_, by hydrolysis and condensation reactions. The organic bridges R offer a broad spectrum of functionalities that can be incorporated into the porous framework [8]. In addition to that the organic bridge R presents reactive centers and hence a variety of reactions are possible for the further modification of the PMO with a large array of desired groups. This expands the range of applications in, for example, optical gas sensing, catalysis, chromatography, separation and nanotechnology [9]. As is typical for mesoporous materials, the pore walls of PMO materials are in most cases X-ray-amorphous. However, some PMO materials offer π–π interactions between the organic bridges in the walls leading to a crystal-like ordering of the organic bridges R within the pore walls, such as in the cases of benzene [10], ethene [11], divinylbenzene [12] or biphenyl [13]. However, direct experimental proofs for this molecular-scale periodicity are only rarely given.

Gas sorption [14], wide-angle X-ray scattering (WAXS), i.e., powder X-ray diffraction (XRD), and IR spectroscopy [15] are commonly used as analysis tools for porous materials in order to determine the structure, surface area and functionalities of the inorganic–organic materials. In addition inverse gas chromatography is quite often used to measure retention volumes in mesoporous silica gels or dispersive surface energies in glasses, for example [16–17]. However, these analysis methods have drawbacks for the analysis of the molecular-scale periodicity of sophisticated materials such as PMOs. They provide information on pore ordering and pore sizes but fail regarding the determination of functional groups within the pores of the host material; IR on the other hand can easily identify functional groups but is limited in its ability to analyze the local distribution of them. Combined techniques help to overcome these limits. Morell et al. studied the formation process of a mesoscopically ordered biphenylene-bridged organosilica with crystal-like pore walls by in situ synchrotron SAXS/WAXS, and demonstrated that the formation of periodicity occurs simultaneously with the formation of the mesopores as the result of a cooperative process [18]. Another advanced technique is the in situ combination of small-angle scattering of neutrons or X-rays (SANS or SAXS) with isothermal gas adsorption [19]. Based on contrast matching, which was introduced by Bragg et al. in 1952 [20], the intensities of the X-ray or neutron scattering reflections of the ordered porous solids are altered if the pores are filled with suitable gases. The degree of matching, however, depends strongly on the chemical nature, i.e., the electron density for SAXS or the neutron scattering length density (SLD) for SANS, of the pore walls in relation to the adsorbed gas. Thus, already small changes at the interface between the host material (the adsorbent) and the adsorbate can be monitored directly [21]. The fact that scattering in SANS measurements takes place at the nucleus, renders it superior compared to SAXS. In SANS there are multiple possibilities in the choice of the adsorbate (e.g., nitrogen, hydrocarbons, water, benzene, etc.), allowing tailored contrast-matching experiments [21–24] and providing insight into both the sorption mechanism, i.e., micropore filling, formation of nitrogen layers and capillary condensation [23–24], and the structural properties. Often adsorbates are favored in which hydrogen atoms can be isotopically substituted by deuterium atoms and, hence, the coherent scattering length of the condensed gas or vapor can be adjusted easily to match that of the solid host material [21]. For example, Tun et al. showed, by in situ adsorption of suitable mixtures of H_2_O/D_2_O on Si-MCM-41 in a SANS equipment, that the resulting population of water in the cylindrical pores after desorption at room temperature is described by a radial function with a minimum at the center and a maximum near the pore wall [19]. By combining SANS with in situ H_2_O/D_2_O (42:58) adsorption at 298 K we could show that functionalization of Si-MCM-41 with SO_3_H-groups by grafting, leads to an inhomogeneous distribution of the groups in the pores, resulting still in strong contrast matching. Samples functionalized by co-condensation, however, exhibited almost no contrast matching, proving a very homogeneous distribution of the SO_3_H-groups along the channels and explaining the observed higher proton conductivities compared to grafted samples [25].

**Scheme 1:**
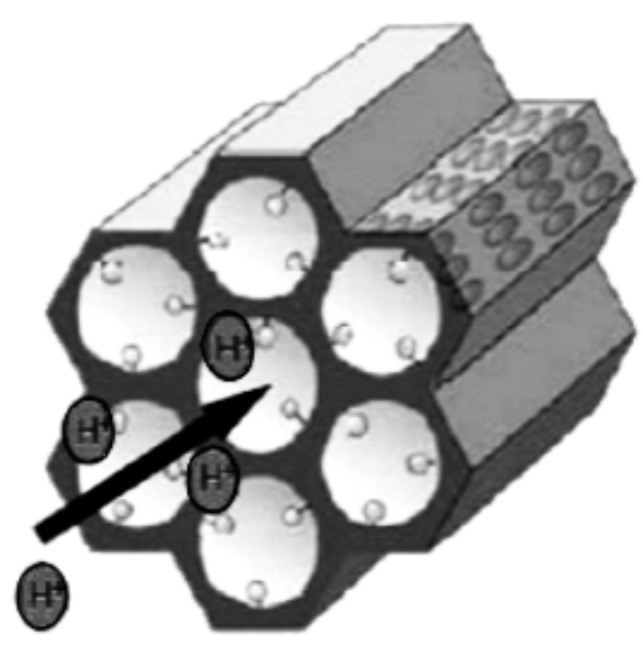
Illustration of the hexagonal pore arrangement of a functionalized benzene-PMO with the benzene rings in the pore walls and the sulfonate or sulfonic acid groups pointing into the channels.

To the best of our knowledge there is only one report in the literature, by Mascotto et al., in which peptide-functionalized benzoic acid PMO materials were investigated by SAXS/SANS with in situ gas adsorption. The authors observed a complete contrast matching with CH_2_Br_2_ and proved that the organic bridges R in the walls as well as the functional groups are homogeneously distributed [26]. However, they did not study the crystal-like ordering of the material. In order to get deeper insights into the crystal-like character of PMO materials with π–π-interacting bridges, we performed in situ SANS/adsorption experiments also at *q*-values higher than 0.5 and could, for the first time, directly prove the molecular-scale periodicity. Because the SLDs of N_2_ in the condensed state at 77 K and of SiO_2_ (also used as a base for PMOs) are almost equal [25], nitrogen is as well a very suitable adsorbate. Compared to those with H_2_O/D_2_O mixtures, adsorption experiments with N_2_ are easier to perform and, in particular, require less time to reach the thermodynamic adsorption equilibrium. Furthermore, pristine benzene-PMO was subsequently functionalized with SO_3_H-groups; such material shows a high proton conductivity [27] and has potential to create proton-conducting hybrid membranes for applications in fuel cells [28–29], electrodialysis for water purification [30], or photoelectrochemical cells for water splitting with solar light in order to separate the generated H_2_ from the simultaneously formed O_2_ [31–32]. Based on functionalized mesoporous silica (e.g., Si-MCM-41-SO_3_H) and different polymer materials (e.g., Nafion^®^ or polysiloxanes) such hybrid membranes have already been realized [28–29]; PMOs, however, may be superior compared to MCM-41 due to their higher hydrothermal stability. The functionalization of the benzene-PMO was realized by grafting, using surface silanol groups as well as benzene rings to anchor the SO_3_H-groups [27]. Scheme 1 shows a sketch of the studied SO_3_H- or SO_3_^−^ sulfonate groups pointing into the channels and the protons hopping within the channels from one sulfonate group to the next.

## Results and Discussion

**Figure 1:**
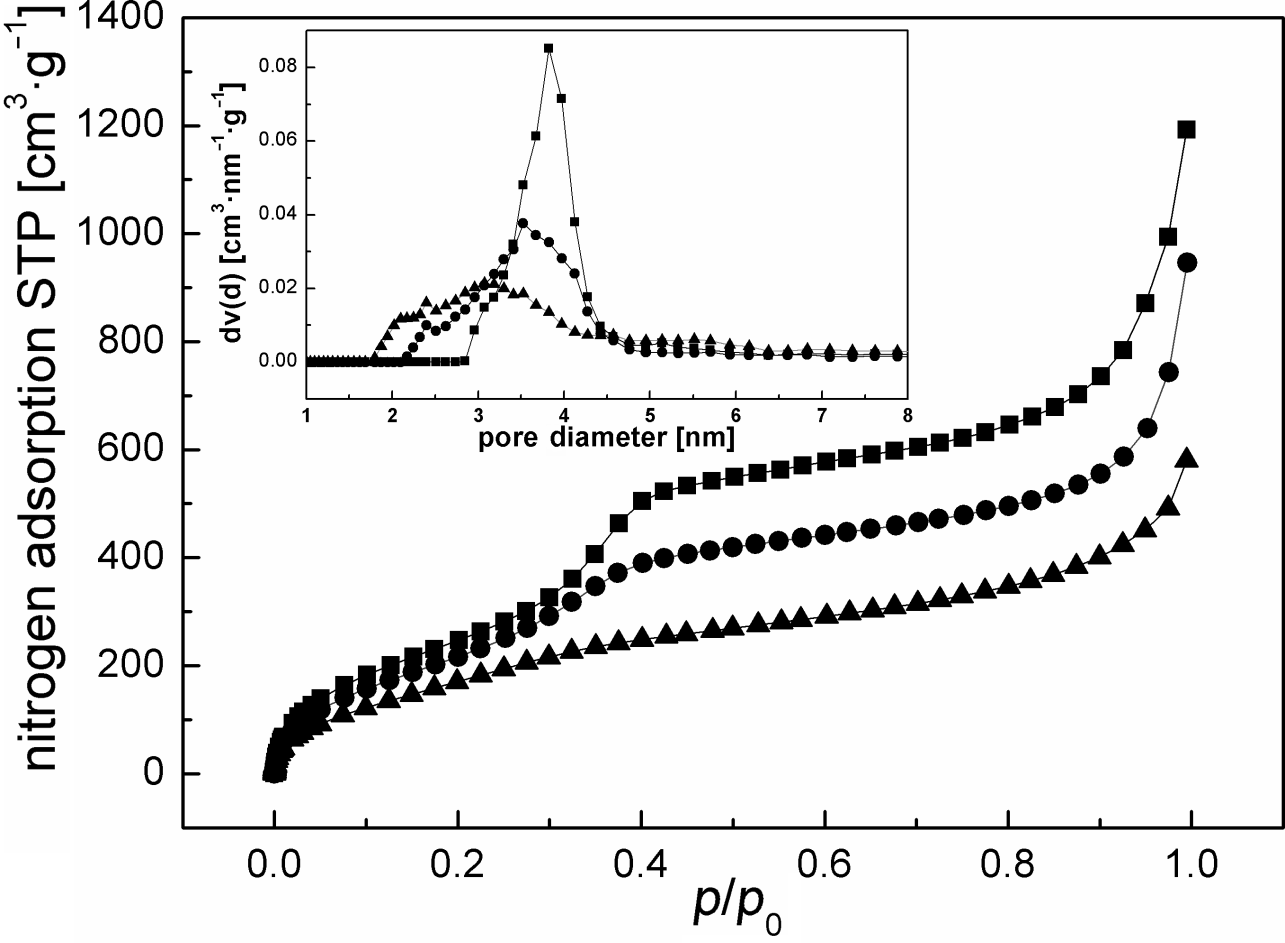
Nitrogen adsorption isotherms and pore diameters (inset); (squares) benzene-PMO, (circles) benzene-PMO-(0.81 mmol SO_3_H·g^−1^) grafted on silanol groups, (triangles) benzene-PMO-(1.65 mmol SO_3_H·g^−1^) grafted on silanol and benzene groups.

Prior to the in situ SANS measurements the synthesized materials were investigated by nitrogen adsorption measurements in order to determine the inner surface areas, the pore volumes and the pore diameters (Figure 1). Nitrogen adsorption on pristine benzene-PMO (squares) exhibits type IV isotherms [33] showing shapes characteristic for the presence of mesopores; their average diameters were determined by DFT-based analysis [34] to be about 3.8 nm (Figure 1 inset). At low relative pressures the amount of adsorbed gas first rises strongly due to monolayer adsorption, followed by a continuous increase due to multilayer adsorption. A strong increase in the adsorbed amount is found at relative pressures *p*/*p*_0_ of 0.2 to 0.42 due to capillary condensation of nitrogen in the mesoporous channels. The sudden increase in the adsorption (*p*/*p*_0_ > 0.9) for all the samples does not result from structural mesoporosity, but is caused by a condensation of nitrogen in the interstitial space between particles (void spaces) and cracks inside the particles. Due to the partial filling of the structural mesochannels by anchoring propyl-SO_3_H groups on the silanol groups of the PMO, the surface area, pore volume and pore diameter of benzene-PMO-(0.81 mmol SO_3_H·g^−1^) (Figure 1, circles) decrease compared to the pristine host material from 952.22 m^2^·g^−1^ to 803.66 m^2^·g^−1^, from 0.846 cm^3^·g^−1^ to 0.752 cm^3^·g^−1^ and from 3.8 nm to 3.5 nm, respectively.

If grafting is performed at the silanol groups as well as the benzene rings, resulting in a total loading of 1.65 mmol SO_3_H per gram as determined by measuring the ion exchange capacity (IEC), the changes in the texture properties for modified benzene-PMO are even more pronounced, as documented by a specific surface area of 487.96 m^2^·g^−1^ and a pore volume of 0.452 cm^3^·g^−1^. Due to the partial pore filling by anchoring of the functional groups, the high uniformity of the benzene-PMO pores is lost; in particular, from the sulfonation at the benzene rings an irregular distribution of pore sizes exhibiting now diameters in the range of 2–4 nm (triangles, inset in Figure 1) results. It is notable that the obvious difference in the isotherms of both functionalized PMO materials is not only explained by the higher loading but occurs especially due to the harsh acidic conditions needed for sulfonation of the benzene rings. However, the mesoporous structure with hexagonal pore ordering is still well developed, as shown below by SANS measurements. To clarify the general influence of the preparation conditions on the pore size distribution, pure benzene-PMO materials were also treated, either with sulfuric acid for 12 h under reflux or with H_2_O_2_ (48 h). Supporting Information File 1 shows the nitrogen isotherms taken before and after the different treatments, demonstrating that especially the strong acidic conditions destroy parts of the regular mesoporous network.

**Figure 2:**
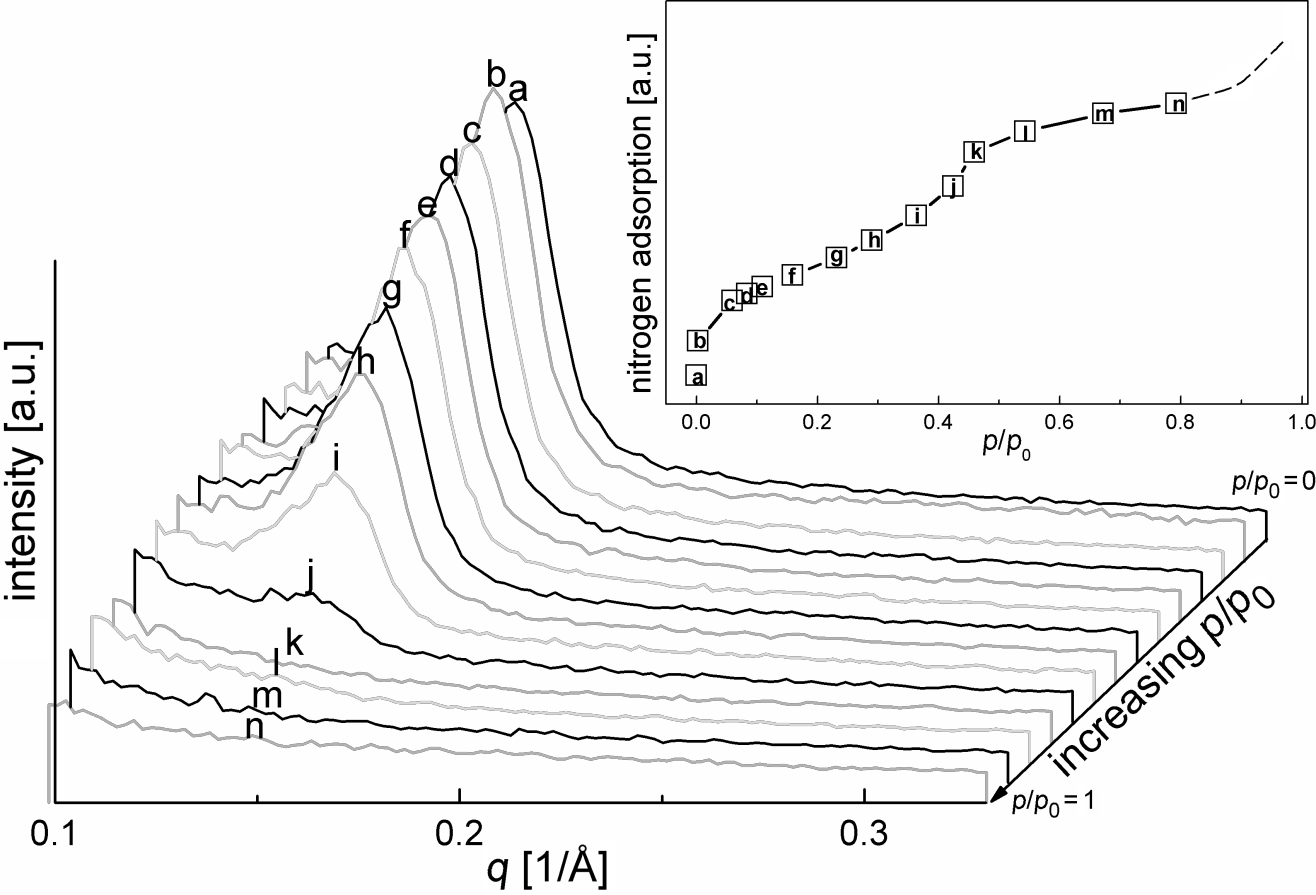
Neutron diffraction patterns of benzene-PMO at small *q* values and different amounts of adsorbed nitrogen.

Figure 2 displays characteristic changes of the neutron scattering intensity for pristine benzene-PMO at different stages of nitrogen adsorption. Since no characteristic peak is shifted in position, indicating that the periodic sequence of pores (i.e., the center–center distance) remains unchanged during the adsorption, the waterfall diagram was chosen for an easier interpretation of the intensity changes in the measured SANS patterns. If the mesopores are empty at *p*/*p*_0_ = 0, the presence of a hexagonal array of mesopores is indicated by a strong (100) neutron scattering peak at *q* = 0.14 1/Å. This observation of only one reflection is in agreement with XRD measurements [5–7,35]. The intensity of this reflection is widely preserved as long as no capillary condensation takes place in the pores. During capillary condensation (starting at point g of the inserted isotherm in Figure 2 and mostly complete at point k) the pores are more and more filled with nitrogen leading almost to a complete disappearance of scattering intensity. After complete pore filling at *p*/*p*_0_ = 0.5 the neutron diffraction signal vanishes fully. This complete contrast matching suggests that the SLD of the benzene-PMO framework, built-up from SiO_2_ and benzene, is quite similar to that of nitrogen in the condensed phase.

The signal intensity *I*(*q*) in SANS for porous materials is proportional to the square of the difference between the SLD of the host material ρ_1_ and the empty pores ρ_2_.

[1]
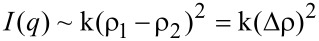


[2]
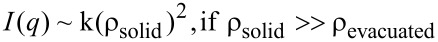


with k being a constant depending on P(*q*), which is a form factor of the pores that is determined by the shapes of the individual pores, and S(*q*) which results from the correlation function of the pores [36]. The SLDs of amorphous silica (ρ_SiO2_ = 3.43·10^10^ cm^−2^) and liquid nitrogen at 77 K (ρ_N2_ = 3.23·10^10^ cm^−2^) are almost equal [37]. However, the mesoporous framework of benzene-PMO consists not only of SiO_2_ but also of organic benzene groups. Thus, since no literature data is yet available for PMO materials, the exact scattering length density must be calculated for further interpretation. The scattering potential of the nucleus of an individual atom is described by the scattering length *b* indicating neutron–nucleus interaction. The scattering length density ρ_SLD_ is defined by the sum of the scattering lengths of all atoms that are involved in the molecular structure, divided by the molecular volume:

[3]
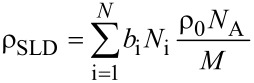


*N*_A_ is the Avogadro constant, *M* is the mass of the used formula unit, e.g., Si_2_O_2_C_6_H_4_ (*M* = 164 g·mol^−1^) for the SLD calculation for pure benzene-PMO, *b*_i_ is the scattering length of atom i, *N*_i_ is the number of atoms of type “i” in the formula unit and ρ_0_ is the density [38–39]. For the SLD calculation of the present material the atomic scattering lengths of *b*_C_ = 6.648 fm, *b*_H_ = −3.741 fm, *b*_Si_ = 4.151 fm, *b*_S_ = 2.874 fm and *b*_O_ = 5.805 fm were used [38]. To determine the density ρ_0_ of each mesoporous material the Archimedean principle was used; the results are given in Table 1. The SLD for 1.65 mmol SO_3_H·g^−1^ functionalized benzene-PMO was calculated by considering, in the formula unit, that the mesoporous surface is altered homogenously by 1.42 mmol SO_3_H·g^−1^ attached on the benzene bridges, as determined by titration with NaOH after the first functionalization step (compare to Experimental section), and more inhomogenously by 0.23 mmol propyl-SO_3_H·g^−1^ attached to silanol groups. The approximate formula of a unit cell is Si_2_O_2_C_6_H_4_(C_3_H_7_SO_3_)_1/24_(SO_3_H)_1/4_.

For the benzene-PMO functionalized with 0.81 mmol SO_3_H·g^−1^ by grafting only at the silanol groups, the situation is more complex, since it must be expected, based on results obtained for grafted samples by different methods [25,34], that the functional groups are distributed inhomogenously and are located mainly at the pore mouths. Under this assumption, in which the functional groups do not reach the inner pore surface, the SLD is only influenced by an altered density but not by the scattering lengths, resulting in a value of 3.84·10^10^ cm^−2^. However, to examine the effect of functional group distribution on the SLD, it was also calculated for the same sample assuming that the functional groups are distributed homogenously and, hence, must be considered in the formula unit, i.e., Si_2_O_2_C_6_H_4_(C_3_H_7_SO_3_)_1/7_. The result given in parentheses in Table 1 shows that the difference in the SLD is not very significant.

**Table 1:** Neutron scattering length densities (SLDs) and densities of benzene-PMO, the two SO_3_H-functionalized benzene-PMOs and nitrogen.

sample	SLD [10^10^·cm^−2^]	density [cm^3^·g^−1^]

benzene-PMO	3.62	2.38
benzene-PMO-(0.81 mmol SO_3_H·g^−1^), grafted on silanol groups	3.84–(3.91)	2.41
benzene-PMO-(1.65 mmol SO_3_H·g^−1^), grafted on silanol and benzene groups	4.55	2.69
nitrogen	3.23	0.807

As explained in Equation 1 and Equation 2, for further interpretation the assumption of a two-phase system was made, in which each phase differs only in its SLD although it may consist of several compounds. Phase one (relating to ρ_1_ in Equation 1) consists of the benzene-PMO framework and condensed nitrogen, because the SLDs of the pure benzene-PMO and liquid N_2_ are almost equal, and phase two consists of empty pores with a SLD of zero (relates to ρ_2_ in Equation 1). Both phases exhibit a different SLD for all stages of adsorption. As long as the assumption for ρ_1_ of comparable SLDs for the framework and the filled pores holds, the reflections of benzene-PMO are erased in SANS measurements if all pores are filled with N_2_ and no inaccessible pores remain empty [23]. However, also for completely filled pores a diffraction signal can remain, if the SLD of the framework is altered from that of the adsorbate, i.e., the assumption for ρ_1_ does not hold. As already shown in Table 1 in the case of benzene-PMO-(1.65 mmol SO_3_H·g^−1^), grafted on the silanol groups and the benzene bridges, ρ_1_ for the framework strongly increases due to the introduction of the functional groups. Thereby, the SO_3_H-groups directly at the benzene rings have a much stronger effect than the propyl-SO_3_H groups at the silanol groups.

Small amounts of adsorbed nitrogen in pristine benzene-PMO show significant peculiarities as shown by the SANS curves in Figure 2. The intensity of the main reflection at *q* = 0.14 1/Å increases at a relative pressure of *p*/*p*_0_ = 0.06 due to the filling of voids with condensed nitrogen, which smoothes the rough inner pore surfaces, i.e., the irregularities in the pore wall thickness that may remain after template extraction [19,39]. As a consequence the intensity of the main reflection increases since the periodicity is improved. Further adsorption of nitrogen results in a continuous decrease of the neutron scattering reflection at *q* = 0.14 1/Å, in agreement with Equation 1 and the assumption of a two-phase system. Under capillary condensation conditions, i.e., at relative pressures *p*/*p*_0_ > 0.26, the SANS reflection intensity *I*(*q*) drops drastically due to N_2_ condensation in the pores. After complete capillary condensation at a relative pressure of *p*/*p*_0_ ≈ 0.50 scattering signals at small *q*-values are no longer observed (Figure 2).

**Figure 3:**
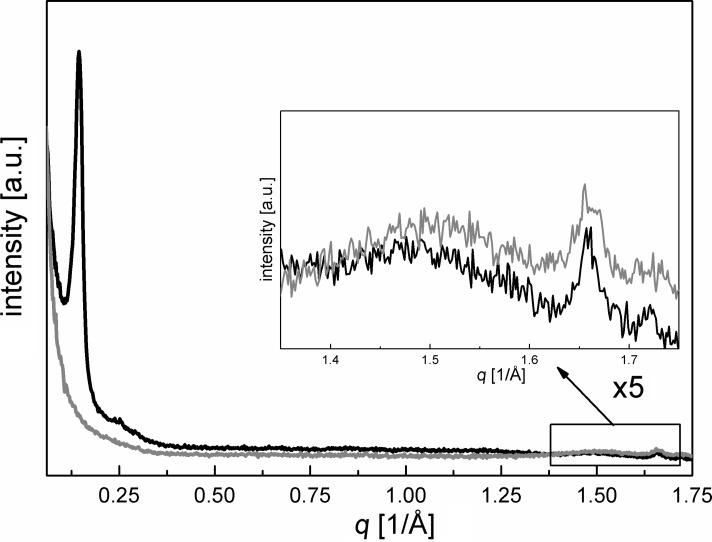
Neutron diffraction patterns of benzene-PMO at small and high *q* values for empty (black curves) and nitrogen-filled pores (gray curves).

Because of the homogenous incorporation of organic groups as bridges between two Si atoms in the mesoporous framework, Inagaki and co-workers found WAXS reflections at high 2θ angles for mesoporous benzene-PMO [5]. Figure 3 shows that, analogous to the XRD measurements, neutron scattering exhibits one fairly sharp reflection at *q* = 1.66 1/Å and a very broad reflection at *q* = 1.44 1/Å, resulting from the periodic arrangement of the benzene groups along the channel direction. In contrast to the totally disappearing reflection at *q* = 0.14 1/Å, representing the long-range order of the mesopores and thus in general the density contrast between wall and pore, these reflections at *q* = 1.44 1/Å and *q* = 1.66 1/Å, reflecting the molecular-scale periodicity in the walls, remain almost unchanged after complete filling of the pores with nitrogen (inset Figure 3). This proves undoubtedly the crystal-like structure of the pore walls of porous PMO materials, and underlines the results found by WAXS measurements [5,7,18].

**Figure 4:**
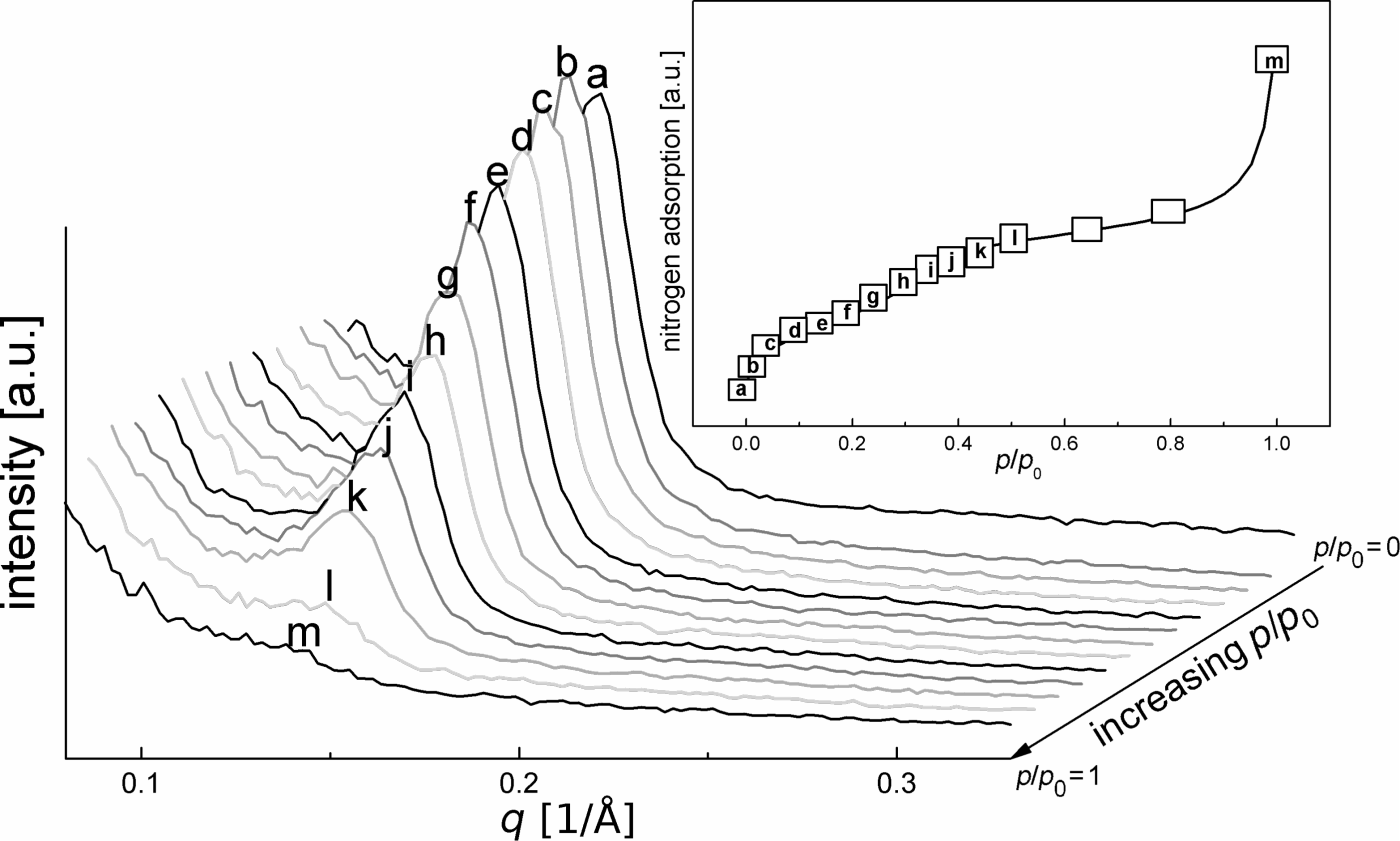
Neutron diffraction patterns of functionalized benzene-PMO-(0.81 mmol SO_3_H·g^−1^) grafted on silanol groups at small *q* values at different stages of adsorbed nitrogen.

Figure 4 shows neutron scattering curves of functionalized mesoporous benzene-PMO-(0.81 mmol SO_3_H·g^−1^) synthesized by anchoring SO_3_H groups via propyl chains onto silanol groups, in dependence on the equilibrium partial pressures of nitrogen adsorbed to the pores. In agreement with results from nitrogen adsorption (Figure 1) the presence of the main reflection peak in the obtained neutron diffraction pattern in vacuum confirms that the mesoporous structure of benzene-PMO-(0.81 mmol SO_3_H·g^−1^) is preserved after the grafting procedure. With continuous nitrogen adsorption the sample exhibits analogous results to pristine benzene-PMO (Figure 2), as the intensity of the main signal at *q* ≈ 0.14 1/Å decreases only weakly at low adsorption levels. Once condensation takes place at relative pressures of *p*/*p*_0_ ≥ 0.22 (inset in Figure 4) a faster decrease of the main signal in the neutron scattering reflection is obtained. After complete pore filling, the hexagonal pore structure becomes invisible to neutron scattering, indicating that the SLD of the walls of benzene-PMO functionalized with 0.81 mmol SO_3_H, formed according to this synthesis route, does not differ significantly from that of pristine benzene-PMO. This result is in agreement with literature data for comparable Si-MCM-41 materials, showing that grafting with quite bulky silanes, such as MPMS, often leads to an inhomogeneous distribution of functional groups [25,35], as seen also by the low decrease in mesopore volume.

**Figure 5:**
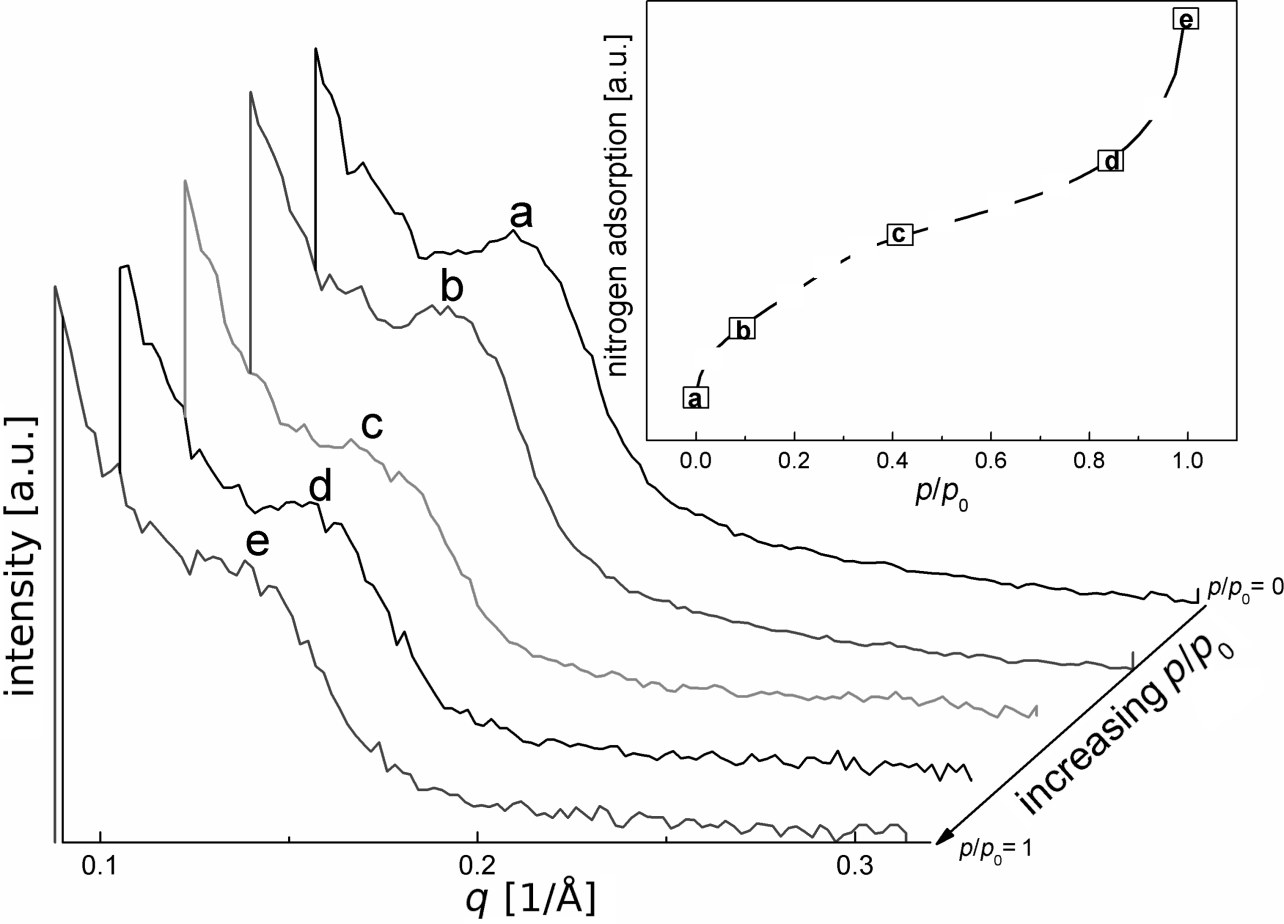
Neutron diffraction patterns of functionalized benzene-PMO-(1.65 mmol SO_3_H·g^−1^) grafted on benzene rings and on silanol groups at small *q* values at different stages of nitrogen adsorption.

Benzene-PMO with a higher degree of functionalization can be achieved by additional modification at the benzene rings. Thus, a maximum SO_3_H loading with an IEC of 1.65 mmol_H+_·g^−1^ was achieved. The neutron scattering reflection found at low *q* values in vacuum for this material (Figure 5) confirms the existence of ordered mesoporous structure, as also found by nitrogen adsorption measurements (compare Figure 1). However, the strongly acidic conditions present during this functionalization procedure (compare to Supporting Information File 1) unambiguously reduce the quality of the mesoporous structure, as indicated by a broadening of the SANS signal and a decrease in intensity (Figure 5) compared to that of the sample functionalized only at the silanol groups. Nevertheless, adsorption of N_2_ alters the reflection intensity and, thus, allows conclusions on the chemical nature of the mesopores. Again, the intensity of the main reflection increases slightly when only a small amount of nitrogen is adsorbed (Figure 5, curve b). Then it decreases slightly with more nitrogen adsorption. However, only a maximum decrease of 21% in intensity is achieved after capillary condensation at relative pressures *p*/*p*_0_ > 0.4 and the reflection peak remains clearly observable (Figure 5, curves d and e). Thus, complete filling of the mesopores does not much alter the SANS patterns. Again the reflections at *q* = 1.66 and 1.44 1/Å resulting from the periodic arrangement in the walls are still present and show, as expected, no changes in intensity after complete pore filling (Supporting Information File 2).

Hence, only weak matching of the neutron diffraction signal was found for benzene-PMO-(1.65 mmol SO_3_H·g^−1^) with a maximum loading of functional groups. This indicates that by functionalization, at the silanol groups as well as the benzene rings, at least about 80% of the entire inner pore surface is altered by functional groups, which are, thus, much more homogeneously distributed than in samples only functionalized by grafting at the silanol groups [6–7]. As a result, in almost the entire mesoporous framework, the SLD of the adsorbed nitrogen is different from that of the (modified) pore walls, impeding contrast matching. The homogenous distribution of SO_3_H-groups on the inner surface of the porous materials is not achieved by the reaction of MPMS at silanol groups, which exclusively appear on the first accessible silanol close to the pore mouths, but by the sulfonation at the benzene rings. Modifications at the stable benzene rings are in general kinetically hindered, which ensures that the SO_3_H/H_2_SO_4_ solution used for the functionalization first spreads uniformly in the whole pore network before significant reaction at the benzene rings in the walls takes place. Analogous results were found for homogeneously SO_3_H-modified Si-MCM-41 (co-condensation route) by SANS measurements with in situ adsorption of a H_2_O/D_2_O mixture, for which a composition was used that tunes the SLD to be equal to that of SiO_2_ [26].

## Conclusion

SANS measurements combined with in situ adsorption of nitrogen at 77 K were used to characterize pristine benzene-PMO and different, functionalized benzene-PMO materials prepared by grafting SO_3_H-groups on silanol and at benzene groups. The obtained neutron diffraction patterns demonstrated that N_2_ matches completely all of the signals of pristine benzene-PMO that are caused by the long-range ordering of the mesopores. Simultaneously signals at higher *q* values were not influenced by the pore filling; this gives, in addition to WAXS patterns, another experimental proof for the crystal-like periodicity of the benzene-PMO. In addition, SLD calculations for benzene-PMO, pure and functionalized with SO_3_H-groups, are reported showing that in particular the SO_3_H groups, which are anchored without any spacer directly at the benzene rings, change the neutron scattering behavior of the framework. In SANS contrast matching measurements the neutron diffraction patterns show that small amounts of SO_3_H-groups (0.81 mmol SO_3_H·g^−1^), anchored via propyl linkers mainly at the pore mouths, only negligibly change the SLD, and complete signal matching by N_2_ is still obtained. However, a higher degree of functionalization with SO_3_H-groups (1.65 mmol SO_3_H·g^−1^), anchored at silanol groups as well as at benzene groups on the surface, hinders complete matching indicating a much more homogenous distribution of functional groups (Figure 5). For a grafting process such a fairly homogeneous introduction of the functional groups was not expected, because many authors have reported blocking effects in the functionalization of mesoporous oxides by grafting, due to almost exclusive functionalization at the pore mouths. And indeed, also here a fairly homogeneous incorporation of the sulfonate groups is only found when functionalization is performed at the silanol as well as at the benzene groups. When functionalization is attempted only at the silanol groups, the incorporation is still inhomogeneous as indicated by the complete matching of the SANS signal in Figure 4.

## Experimental

Synthesis of benzene-PMO: Ordered mesoporous benzene-PMO was synthesized by adding 7.49 g octadecyl-trimethylammoniumchloride surfactant to 225 mL distilled water and 14.4 mL NaOH (6 M) at 328 K [1]. After a clear solution was obtained, 9 g 1,4-bis(triethoxysilyl)benzene (BTEB) were added to the surfactant solution at room temperature under vigorous stirring. The mixture was treated ultrasonically for 20 min to disperse the hydrophobic BTEB in the aqueous solution. Afterwards the dispersion was stirred for 20 h at room temperature and kept for 24 h at 368 K under static conditions. The resulting white solid was filtered and washed with water and ethanol. The surfactant was removed by extraction in 250 mL ethanol with 7.5 mL 36% HCl at 353 K for 24 h.

Synthesis of benzene-PMO-SO_3_H, SO_3_H-groups grafted onto silanol groups: 0.5 g benzene-PMO was added to a flask and dried under vacuum for 3 h. Under an argon atmosphere the white powder was suspended in 20 mL dry toluene. Afterwards 1.2 mL 3-mercaptopropyltrimethoxysilane (MPMS) was added and the suspension was heated under reflux up to 383 K. The reaction mixture was stirred for 24 h followed by filtration and washing with toluene and ethanol. In order to obtain SO_3_H-groups the attached SH-groups were oxidized with hydrogen peroxide. Therefore 0.3 g of the MPMS functionalized sample prepared by grafting was suspended in 10 mL of H_2_O_2_ solution (30 wt %) and stirred for 48 h at room temperature. The powder was filtered and washed with ethanol and water. Then the oxidized solid was suspended in 30 mL of a 2 M H_2_SO_4_ solution, stirred for 2 h at room temperature, and then finally filtered and again washed with ethanol and water. The loading with exchangeable H^+^ ions was determined by titration with NaOH (1 M), resulting in 0.81 mmol SO_3_H·g^−1^ when SO_3_H-groups were grafted only on silanol groups.

Synthesis of benzene-PMO-SO_3_H, SO_3_H-groups grafted on benzene rings and on silanol groups: 0.8 g benzene-PMO was dried under vacuum for 3 h. Afterwards 50 mL of a 25% SO_3_/H_2_SO_4_ (oleum) solution were added under an argon atmosphere. The dispersed solution was heated to 378 K and kept at that temperature for 10 h under reflux. The resulting solid exhibiting 1.42 mmol SO_3_H groups per g, as determined by titration with NaOH, was filtered and washed with 3 L water. Thus, the material was functionalized at the benzene rings. Afterwards 0.5 g of the product was additionally functionalized at the silanol groups by grafting with MPMS as described before. Finally, the solid was stirred for 2 h in 30 mL HCl to protonate and form sulfonic acid groups resulting in a maximum loading of 1.65 mmol SO_3_H-groups per gram.

### Characterization

Neutron diffraction patters with in situ gas adsorption were collected at the V1 diffractometer of the cold neutron source (λ = 5.23 1/Å) of the BER-II reactor located at the Helmholtz Center Berlin (HZB). The scattering intensity was collected in the range of *q* = 0.01–0.7 1/Å by varying the detector position at a sample-to-detector distance of 102.52 cm. The scattering vector *q* is defined as *q* = 4π/λ sin(θ), with λ being the wavelength and θ the Bragg angle. The temperature during neutron experiments was 77 K. Before the measurement of each neutron scattering pattern, the sample was filled with a certain amount (*n*/*n*_0_) of adsorbent, by applying a gas-adsorption setup, which was connected to the measuring cell and enables a direct in situ SANS measurement for each point of the *p*–*V* adsorption isotherm. SANS measurements were only performed after a constant cell pressure was obtained, which ensures an adsorption equilibration. Nitrogen adsorption isotherms at 77 K were determined on a Quantachrome Autosorb 3 apparatus. Prior to each adsorption measurement, the samples were outgassed at 433 K for 24 h. The Brunauer–Emmett–Teller (BET) method was used to determine the specific surface area. The pore volumes and pore diameters were calculated according to density functional theory (DFT) [34]. In order to determine the density of pure and SO_3_H-functionalized benzene-PMO materials the Archimedean principle was used. Thus, each sample was hand-pressed to a pellet and dried in an oven at 373 K for 48 h before being weighed in air. After that, each sample was soaked in the impregnation solution prior to another weighing with the aid of a hydrostatic balance. Hereby, it is important that the sample was immersed completely into the impregnation solution.

## Supporting Information

The Supporting Information features nitrogen isotherms and the pore diameter of benzene-PMO taken before and after the different acidic treatments for functionalization (Supporting Information File 1). SANS diffraction patterns of functionalized benzene-PMO with 1.65 mmol SO_3_H groups grafted on benzene rings and silanol groups after complete pore filling with nitrogen are shown in Supporting Information File 2.

File 1 Nitrogen adsorption isotherms and pore diameters (inset); (triangles) benzene-PMO, (circles) benzene-PMO stirred in conc. H_2_SO_4_ for 12 h, (squares) benzene-PMO stirred in 30% H_2_O_2_ for 48 h.

File 2 Neutron diffraction patterns of functionalized benzene-PMO with 1.65 mmol SO_3_H groups grafted on benzene rings and on silanol groups at small *q* values after complete pore filling with nitrogen.
