# Supplementary figures and images for: Distribution of functional groups in periodic mesoporous organosilica materials studied by small-angle neutron scattering with in situ adsorption of nitrogen
Source: Beilstein J Nanotechnol. 2012 May 30;3:428–37. doi: 10.3762/bjnano.3.49 (PMC3388367; doi:10.3762/bjnano.3.49)

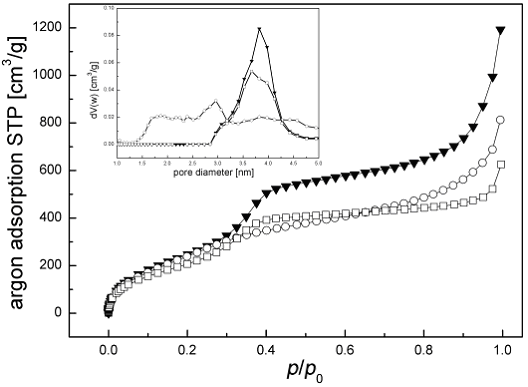

Supplement: File 1 — Nitrogen adsorption isotherms and pore diameters (inset); (triangles) benzene-PMO, (circles) benzene-PMO stirred in conc. H2SO4 for 12 h, (squares) benzene-PMO stirred in 30% H2O2 for 48 h. [file Beilstein_J_Nanotechnol-03-428-s001.png]

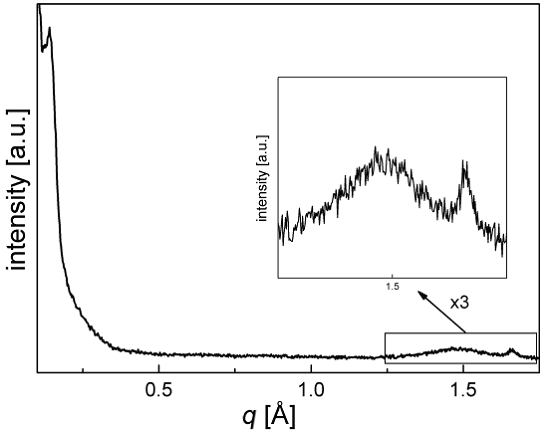

Supplement: File 2 — Neutron diffraction patterns of functionalized benzene-PMO with 1.65 mmol SO3H groups grafted on benzene rings and on silanol groups at small q values after complete pore filling with nitrogen. [file Beilstein_J_Nanotechnol-03-428-s002.png]
